# Recent development of machine learning-based methods for the prediction of defensin family and subfamily

**DOI:** 10.17179/excli2022-4913

**Published:** 2022-05-05

**Authors:** Phasit Charoenkwan, Nalini Schaduangrat, S. M. Hasan Mahmud, Orawit Thinnukool, Watshara Shoombuatong

**Affiliations:** 1 Modern Management and Information Technology, College of Arts, Media and Technology, Chiang Mai University, Chiang Mai, Thailand, 50200; 2 Center of Data Mining and Biomedical Informatics, Faculty of Medical Technology, Mahidol University, Bangkok, Thailand, 10700; 3 Department of Computer Science, American International University-Bangladesh (AIUB), Kuratoli, Dhaka 1229, Bangladesh

**Keywords:** defensins, sequence analysis, bioinformatics, classification, machine learning, feature selection

## Abstract

Nearly all living species comprise of host defense peptides called defensins, that are crucial for innate immunity. These peptides work by activating the immune system which kills the microbes directly or indirectly, thus providing protection to the host. Thus far, numerous preclinical and clinical trials for peptide-based drugs are currently being evaluated. Although, experimental methods can help to precisely identify the defensin peptide family and subfamily, these approaches are often time-consuming and cost-ineffective. On the other hand, machine learning (ML) methods are able to effectively employ protein sequence information without the knowledge of a protein's three-dimensional structure, thus highlighting their predictive ability for the large-scale identification. To date, several ML methods have been developed for the *in silico* identification of the defensin peptide family and subfamily. Therefore, summarizing the advantages and disadvantages of the existing methods is urgently needed in order to provide useful suggestions for the development and improvement of new computational models for the identification of the defensin peptide family and subfamily. With this goal in mind, we first provide a comprehensive survey on a collection of six state-of-the-art computational approaches for predicting the defensin peptide family and subfamily. Herein, we cover different important aspects, including the dataset quality, feature encoding methods, feature selection schemes, ML algorithms, cross-validation methods and web server availability/usability. Moreover, we provide our thoughts on the limitations of existing methods and future perspectives for improving the prediction performance and model interpretability. The insights and suggestions gained from this review are anticipated to serve as a valuable guidance for researchers for the development of more robust and useful predictors.

## Introduction

Nearly all living species comprise of host defense peptides called defensins, that are crucial for innate immunity. Defensins are considered as a part of the antimicrobial protein family and are rich in cysteine. Furthermore, defensins offer assistance to cells in combating bacterial (Menendez and Finlay, 2007[43]), viral (Wilson et al., 2013[56]) and fungal infections (Parisi et al., 2019[44]; Sathoff and Samac, 2019[46]) by destroying the structural integrity of bacterial cell membranes (Bun Ng et al., 2013[5]; De Coninck et al., 2013[19]; de Oliveira Dias and Franco, 2015[21]). Precisely, defensins bind to the microbial cell membrane forming a pore-like channel in the membrane which cause ions and nutrients to leak through biphasic permeabilization (Jarczak et al., 2013[26]). Further evidence suggests that a predisposition to diseases (Kim et al., 2015[31]) may be caused by an imbalance or reduction (Albrethsen et al., 2005[2]) of defensins in various organisms. 

**Figure 1:**
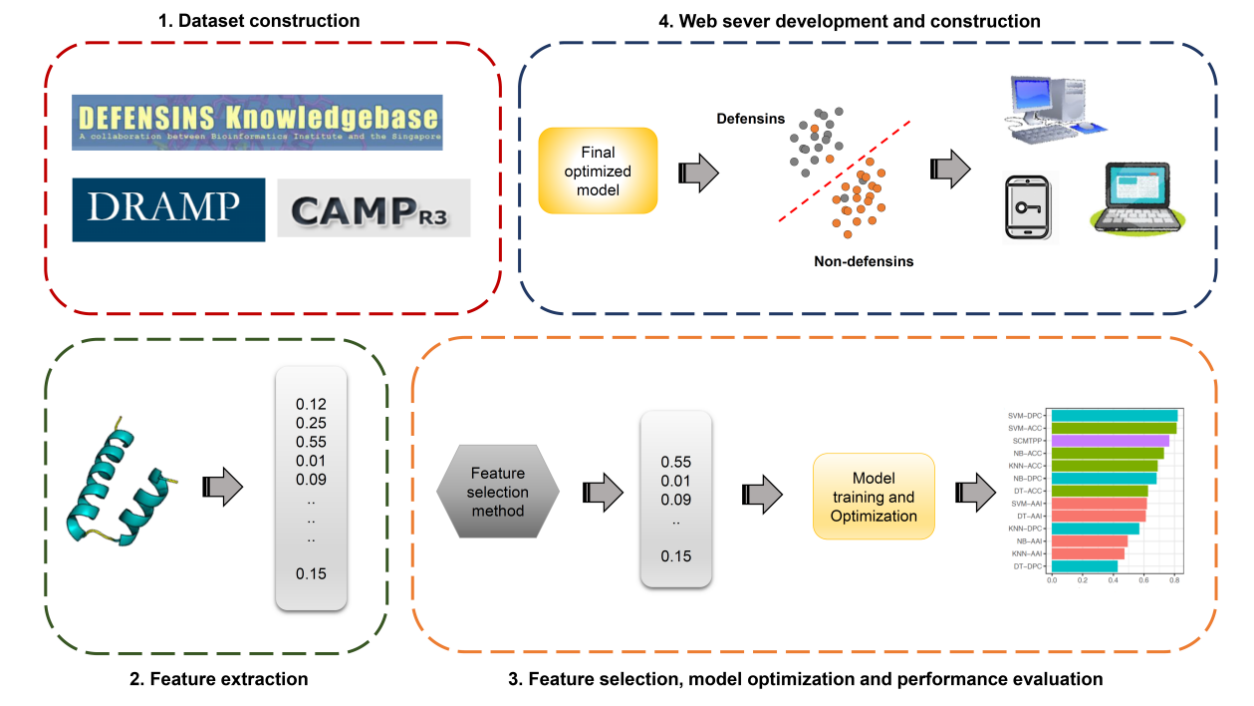
The general machine learning framework of the prediction of defensins and their family/subfamily

In addition, defensins are shown to exhibit a wide range of key applications in various industries thus, highlighting the importance of their design to fit specific needs (Whiston et al., 2017[55]). Nevertheless, conventional experimental approaches, such as nuclear magnetic resonance (de Medeiros et al., 2010[20]), are often time-consuming and not cost-effective. On the other hand, there is an increase in the number of new proteins sequenced by next-generation sequencing techniques. As a result, a large number of novel defensin candidates can potentially be found in these proteins. Thus, it is desirable to rapidly and accurately identify defensins from large-scale proteins. Previously, machine-learning (ML) methods were naturally selected to conduct a large-scale identification and prediction of several proteins and peptides (Li et al., 2015[35]; Lin et al., 2010[37], 2019[38]; Lv et al., 2020[40]; Su et al., 2018[49]; Xu et al., 2019[57]; Zhang et al., 2021[58]; Zulfiqar et al., 2021[59]). These approaches are able to effectively employ protein sequence information without the knowledge of the protein's three-dimensional structure. Furthermore, the general machine learning framework used for the prediction of defensins involves four major steps as summarized in Figure 1(Fig. 1), including, the preparation of training and independent test datasets, feature extraction, feature optimization, and model development and evaluation. Currently, there are six state-of-the-art computational approaches that have been developed for the *in silico* prediction of defensins, including, Karnik's method (Karnik et al., 2009[29]), ID_RAAA (Zuo and Li, 2009[62]), Defensinpred (Ramya Kumari et al., 2012[45]), iDPF-PseRAAAC (Zuo et al., 2015[61]), iDEF-PseRAAC (Zuo et al., 2019[60]) and DEFPRED (Kaur et al., 2021[30]) as summarized in Table 1(Tab. 1) (References in Table 1: Karnik et al., 2009[29]; Kaur et al., 2021[30]; Ramya Kumari et al., 2012[45]; Zuo and Li, 2009[62]; Zuo et al., 2015[61], 2019[60]). 

We categorize these computational methods in Table 1(Tab. 1) into two groups according to their predictive applications. The first group is comprised of computational methods developed for the *in silico* prediction of defensins, which make up two out of six existing methods (i.e., Karnik's method (Karnik et al., 2009[29]) and DEFPRED (Kaur et al., 2021[30])). The second group is focused on those computational methods which have been developed for the *in silico* prediction of the defensin peptide family and subfamily, and comprise of four out of the six existing methods (i.e., ID_RAAA (Zuo and Li, 2009[62]), Defensinpred (Ramya Kumari et al., 2012[45]), iDPF-PseRAAAC (Zuo et al., 2015[61]) and iDEF-PseRAAC (Zuo et al., 2019[60])).

Motivated by the above-mentioned considerations, we provide a comprehensive comparison and analysis of the current state-of-the-art computational methods. Major contributions of this review article could be summarized as follows: (i) to the best of our knowledge, this article provides the first comprehensive review on the development of computational approaches for the *in silico* identification of the defensin peptide family and subfamily; (ii) we have provided several important aspects that play a crucial role for the development of reliable and stable prediction models, covering, their dataset quality, feature encoding methods, feature selection schemes, ML algorithms, cross-validation methods and web server availability/usability and (iii) we have discussed the limitations as well as the advantages and disadvantages of existing methods and provided future perspectives for improving the prediction performance and model interpretability.

## Materials and Methods

### General machine learning framework of the predictions of defensins and their family/subfamily

Until now, a number of computational approaches for *in silico* prediction of defensins and their family/subfamily have been developed (Karnik et al., 2009[29]; Kaur et al., 2021[30]; Ramya Kumari et al., 2012[45]; Seebah et al., 2007[47]; Zuo and Li, 2009[62]; Zuo et al., 2015[61]). The general machine learning framework used for the prediction of defensins involves four major steps as summarized in Figure 1(Fig. 1). The first step is the preparation of training and independent test datasets. The training and independent test datasets are used for cross-validation and model validation purposes, respectively. The second step is the feature extraction. There are many feature encoding schemes that are used to encode variable-length proteins and peptides into fixed-length feature vectors. However, using the original feature dimensions might include irrelevant/redundant information as well as require additional computational resources during model optimization. On the other hand, the performance is not robust in many cases. Therefore, the third step is to select a set of important features. The fourth step is to train and evaluate a prediction model. The effectiveness and robustness of the prediction models are assessed on the independent test dataset. Finally, the optimal prediction model is employed to establish a web server.

### Datasets

We reviewed all the datasets used for developing the existing methods (Karnik et al., 2009[29]; Kaur et al., 2021[30]; Zuo and Li, 2009[62]; Zuo et al., 2015[61], 2019[60]). The detailed information of these datasets are provided in Table 2(Tab. 2) (References in Table 2: Karnik et al., 2009[29]; Kaur et al., 2021[30]; Ramya Kumari et al., 2012[45]; Zuo and Li, 2009[62]; Zuo et al., 2015[61], 2019[60]). As seen in Table 2(Tab. 2), the datasets of Zou2015 (Zuo et al., 2015[61]) and Zou2019 (Zuo et al., 2019[60]) derived from the defensins knowledgebase (Seebah et al., 2007[47]) applied a lower CD-HIT threshold of 0.8 in order to exclude all homologous sequences. For the Zou2015 dataset (Zuo et al., 2015[61]), it contained 333 defensin proteins, which were classified into 60 insect defensins, 34 invertebrate defensins, 42 plant defensins, 157 vertebrate defensins and 40 unclassified defensins. Among the 157 vertebrate defensins, they were also classified as alpha-, beta- and theta-defensins. In the case of the Zou2019 dataset (Zuo et al., 2019[60]), it contained 333 defensin proteins, which were classified as 60 insect defensins, 31 invertebrate defensins, 42 plant defensins, 157 vertebrate defensins and 38 unclassified defensins.

Recently, Kaur et al. established two up-to-date datasets (Kaur2021 (Kaur et al., 2021[30])) containing a main and alterative datasets. In Kaur2021, the defensin samples were collected from various sources, including literature (Zuo and Li, 2009[62]; Zuo et al., 2015[61], 2019[60]), DRAMP2.0 (Kang et al., 2019[28]) and CAMPR3 (Waghu et al., 2016[53]). The samples in the work of (Kaur et al., 2021[30]) were experimentally validated defensins exhibiting antimicrobial activity and the number of residues were in the range of 10-60. However, sequences containing non-natural or non-standard amino acids (B, J, O, U, X, and Z) were excluded. As a result, a total of 1,036 unique defensins were obtained and used to create the main and alterative datasets. For the main dataset, it contained 1,036 positives and 1,036 negatives, where positives and negatives are experimentally validated defensins and antimicrobial peptides (AMPs), respectively. In case of the alternative dataset, it contained 1,036 positives and 1,054 negatives, where positives and negatives are experimentally validated defensins and selected peptides from Swiss-Prot (UniProt Consortium, 2017[50]), respectively.

### Machine learning algorithms used for the prediction of defensins and their family/subfamily

As can be seen from Table 1(Tab. 1), SVM is the most popular ML algorithm for building computational models in the prediction of defensins and their family/subfamily, used in Defensinpred (Ramya Kumari et al., 2012[45]), iDPF-PseRAAAC (Zuo et al., 2015[61]), iDEF-PseRAAC (Zuo et al., 2019[60]) and DEFPRED (Kaur et al., 2021[30]). In the meanwhile, the RF and ID methods were used to develop Karnik's method (Karnik et al., 2009[29]) and ID_RAAA (Zuo and Li, 2009[62]), respectively. Hereafter, we provide the basic concepts of SVM and RF algorithms.

SVM is a well-known and powerful ML algorithm that is commonly employed to deal with binary classification problems (Vapnik, 1999[51], 2000[52]). In particular, SVM maps the given input features into a higher dimensional space using kernel functions and finds optimal hyperplanes that can separate positive samples from negative samples. To date, there are several kernel functions used for developing SVM classifiers, such as linear function, polynomial function, sigmoid function and gaussian radial basis function (RBF). Amongst the several kernel functions, the RBF kernel is the most commonly used one. In order to enhance the performance of SVM classifiers, a grid search strategy was utilized to optimize the two important aspects of the RBF kernel, including *C* (controls the trade-off between the misclassification rate and margin) and γ (the kernel width parameter). Although SVM often yields satisfactory prediction performances, this method is known as a black-box computation method (Ahmad et al., 2022[1]; Charoenkwan et al., 2021[12]; Li et al., 2021[33]; Wei et al., 2021[54]). 

RF is another powerful and widely employed ML algorithm for dealing with binary classification problems (Charoenkwan et al., 2020[13]; Hasan et al., 2020[23], 2021[24]; Manavalan et al., 2019[41][42]; Su et al., 2020[48]). RF is an ensemble-based method originally introduced by Leo Breiman (2001[4]) that is created by integrating a number of decision trees. Each decision tree consists of a single root node, leaf nodes and a number of intermediate nodes (Breiman, 2001[4]). An if-then rule is derived from the path connecting the root node to the leaf node. As a result, RF is able to provide a collection of if-then rules. Therefore, this method is known as a white-box computation method. In order to enhance the performance of RF classifiers, a grid search strategy was employed to optimize two key parameters: *mtree* (the number of decision trees) and *mtry* (the number of selected features).

### Performance evaluation and evaluation strategy

Here, we employed five commonly used performance measures to comprehensively evaluate and analyze the performance of the six state-of-the-art predictors (Karnik et al., 2009[29]; Kaur et al., 2021[30]; Ramya Kumari et al., 2012[45]; Seebah et al., 2007[47]; Zuo and Li, 2009[62]; Zuo et al., 2015[61]), including ACC, Sn, Sp, MCC and OA. The definitions of these performance measures are defined as follows:


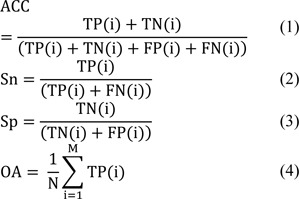


where TP(i), TN(i), FP(i) and FN(i) denote true positives, true negatives, false positives and false negatives of the i^th^ class or the i^th^ family. And, M and N are the number of subsets and the number of samples, respectively.

## Results and Discussion

### State-of-the-art computational approaches for the prediction of defensins

In this section, we conducted a performance comparison for the two existing methods for the prediction of defensins (Karnik's method (Karnik et al., 2009[29]) and DEFPRED (Kaur et al., 2021[30])). The performance comparison results are provided in Table 3(Tab. 3) (References in Table 3: Karnik et al., 2009[29]; Kaur et al., 2021[30]). In 2009, Karnik et al. developed the first ML-based predictor (called Karnik's method (Karnik et al., 2009[29])) to discriminate defensins from non-defensins. The dataset consisting of 238 defensins and 238 non-defensins, was randomly partitioned into train (80 %) and independent test (20 %) datasets. Specifically, Karnik's method was created using the RQA descriptor coupled with RF algorithm by tuning the *mtry* parameter based on the 10-fold cross-validation scheme and the training dataset. 

Recently, Kaur et al. created DEFPRED (Kaur et al., 2021[30]) for specifically discriminating defensins from AMPs (antimicrobial peptides, or non-defensins). DEFPRED was developed using various types of features, including AAC, APAAC, DPC, CTD, PSSM, etc. In their study, the support vector classifier (SVC) coupled with linear kernel penalized with L1 regularization (SVC-L1) method was employed to determine the optimal feature subset. In addition, several ML algorithms were used to develop the models, including RF, SVM, extra tree (ET), logistic regression (LR), k-nearest neighbors (KNN) and multilayer perceptron (MLP). The model that achieved the highest predictive performance was considered as the optimal one (DEFPRED). A user-friendly web sever is publicly available at https://webs.iiitd.edu.in/raghava/defpred/. As seen in Tables 1(Tab. 1) and 3(Tab. 3), DEFPRED outperforms Karnik's method in terms of generalization ability, robustness and utility.

### State-of-the-art computational approaches for the prediction of defensin family and subfamily

As mentioned above, there are four existing methods developed for the predictions of defensins family and vertebrate defensins subfamily, including ID_RAAA (Zuo and Li, 2009[62]), Defensinpred (Ramya Kumari, et al., 2012[45]), iDPF-PseRAAAC (Zuo et al., 2015[61]) and iDEF-PseRAAC (Zuo et al., 2019[60]). The performance comparison results are provided in Tables 4-5(Tab. 4)(Tab. 5). In 2009, Zuo and Li first proposed ID_RAAA (Zuo and Li, 2009[62]), an ID-based approach in conjunction with RAAA descriptor. The ID is a similarity-based approach used for measuring the similarity score of two diversity samples. ID_RAAA was created for the prediction of the defensin family (vertebrate defensins, plant defensins, insect defensins and others) and subfamily (alpha-type, beta-type and theta-type). In the RAAA descriptor, there are two main parameters: different sizes (S = 5, 8, 9, 11, 13, 20) and N-peptide compositions (N = 1, 2, 3). For the prediction of the defensin family, an OA of 91.36 %.was obtained by using a combination of N = 2 and S = 13 as indicated by the jackknife test. The combination of N = 2 and S = 13 still achieved an OA of 94.21 % for the prediction of the defensin subfamily. 

Zuo et al. (2015[61]) introduced iDPF-PseRAAAC, a multi-class SVM predictor coupled with the RAAA descriptor. Zuo's study was proposed to address a small number of samples (Zuo and Li, 2009[62]). As a result, Zuo et al. collected more than 500 defensin proteins from the defensins knowledgebase (Seebah et al., 2007[47]). Then, the CD-HIT threshold of 0.8 was used to exclude sequence redundancy. Finally, Zuo et al. obtained a dataset containing 333 defensin proteins. These defensin proteins could be classified into five families, including insect defensins, invertebrate defensins, plant defensins, vertebrate defensins and unclassified defensins. The SVM classifier coupled with a combination of N = 2 and S = 13 (called iDPF-PseRAAAC) yielded an OA of 99.36 %. In addition, the 10-fold cross-validation results were also performed to assess the predictive ability of iDPF-PseRAAAC. The 10-fold cross-validation and jackknife test results were 83.78 % and 85.59 %, respectively. For the vertebrate defensin subfamily prediction, iDPF-PseRAAAC provided an OA of 98.39 %, while the MCC for the prediction of alpha-type, beta-type and theta-type was 0.97, 0.96 and 0.89, respectively (Table 5(Tab. 5); References in Table 5: Karnik et al., 2009[29]; Zuo et al., 2015[61]).

In 2019, Zuo et al. presented iDEF-PseRAAC (Zuo et al., 2019[60]) by applying SVM algorithm and the F-score method. In iDEF-PseRAAC, it was developed based on a brand-new descriptor (i.e., reduced amino acid resource) containing more than 600 types of features. Their comparative results showed that the DPC of type 5 and cluster 19 (T = 5, C = 19) provided an OA of 91.16 %. To improve the predictive performance, the F-score method was employed to select informative features. Then, the 329 selected informative features were obtained and they achieved an OA of 92.38 %. The SVM classifier in conjunction with the 329 selected informative features was considered as iDEF-PseRAAC in the work of Zuo et al. (2019[60]). In the case of the defensin family prediction, iDEF-PseRAAC gave an OA of 92.38 %. Meanwhile for the vertebrate defensin subfamily prediction, iDEF-PseRAAC gave an OA of 98.79 %, an Sn of 0.99, and an Sp of 0.99.

From Table 2(Tab. 2), it can be observed that, iDPF-PseRAAAC and iDEF-PseRAAC were developed for predicting the five defensin protein families (i.e., insect defensins, invertebrate defensins, plant defensins, vertebrate defensins and unclassified defensins), while ID_RAAA were developed for predicting the four defensin protein families (i.e., insect defensins, plant defensins, vertebrate defensins and unclassified defensins). Therefore, we conducted a performance comparison between iDPF-PseRAAAC and iDEF-PseRAAC in order to make a fair conclusion. As can be seen from Table 4(Tab. 4) (References in Table 4: Karnik et al., 2009[29]; Zuo et al., 2019[60]), iDEF-PseRAAC achieves the best overall performance as compared with iDPF-PseRAAAC for all the five families of defensins in terms of Sn and MCC. To be specific, the MCC of iDEF-PseRAAC was 0.86, 0.64, 0.90, 0.88 and 0.46 respectively, which were 7 %, 9 %, 1 %, 7 % and 23 % higher than that of iDPF-PseRAAAC for insect defensins, invertebrate defensins, plant defensins, vertebrate defensins and unclassified defensins, respectively (Table 4(Tab. 4)). Taken together, iDEF-PseRAAC outperforms ID_RAAA and iDPF-PseRAAAC in terms of predictive performance and robustness.

### Characterization of defensins based on sequence information

**Figure 2:**
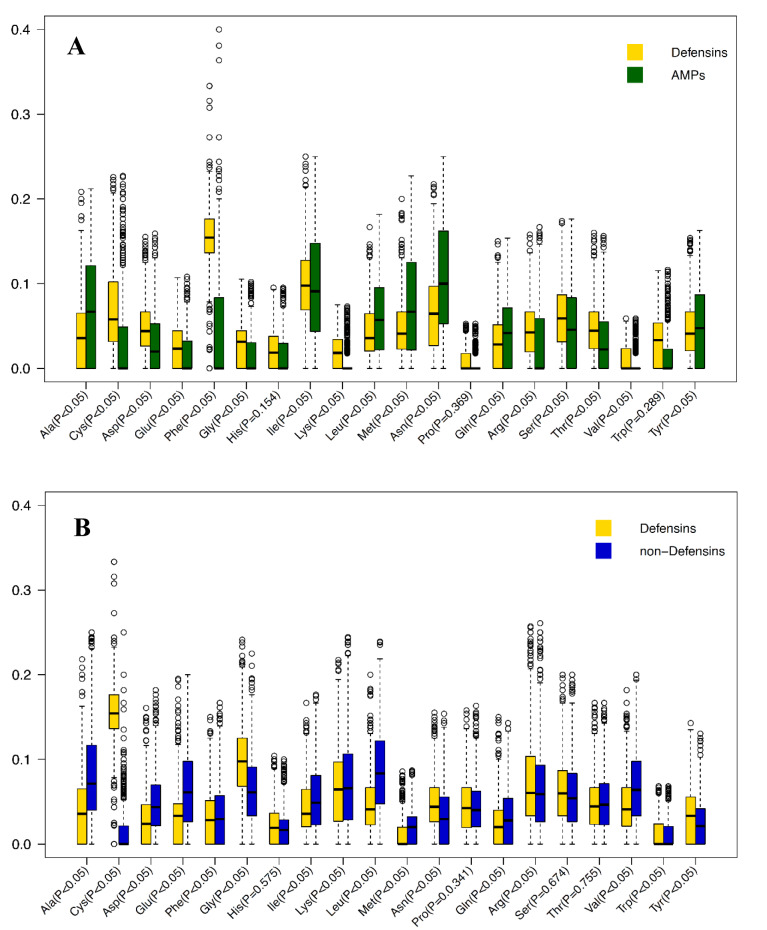
Boxplots of average amino acid compositions of 20 amino acids of Defensins vs AMPs (A) and Defensins vs non-Defensins (B). X- and Y-axes represent 20 amino acids along with their *p*-value and average amino acid composition.

Kaur et al. (2021[30]) provided compositional analysis and preferential position analysis based on the main and alternative datasets. As mentioned above, the main dataset contains 1,036 defensins and 1,036 AMPs, while the alternative dataset contains 1,036 defensins and 1,054 non-defensins. As shown in Figure 2A(Fig. 2), it can be observed that Cys, Asp, Glu, Asn, Arg, Thr and Tyr were found to be abundant in defensins as compared to AMPs, while Phe, Ile, Ala, Lys, Leu were found to be abundant in AMPs as compared to defensins. Interestingly, most of the amino acids were significantly different between the classes at the level of *P* < 0.05, with the exception of His (*P* = 0.154), Pro (*P* = 0.369) and Trp (*P* = 0.289). In addition, Figure 2B(Fig. 2) reveals that Cys, Gly, Arg, and Thr were abundant in defensins as compared to non-defensins, while Asp, Val, Glu, Leu and Ala were abundant in non-defensins as compared to defensins. Furthermore, most of the amino acids were significantly different between the classes at the level of *P* < 0.05, with the exception of His (*P* = 0.575), Pro (*P* = 0.341), Ser (*P* = 0.674) and Thr (*P* = 0.755). Taken together, Cys, Tyr, Arg and Asn might be important amino acids for defensins. In addition, the prevalence of these four amino acids (i.e., Cys, Try, Arg and Asn) are significantly different between defensins and AMPs/non-defensins at the level of *P* < 0.05.

### Web server availability and usability

As can be seen from Table 1(Tab. 1), among the five state-of-the-art computational approaches, four of them were implemented as web servers for the prediction of defensins (DEFPRED (Kaur et al., 2021[30])) and their family/subfamily (Defensinpred (Ramya Kumari et al., 2012[45]), iDPF-PseRAAAC (Zuo et al., 2015[61]) and iDEF-PseRAAC (Zuo et al., 2019[60])). Unfortunately, only two web servers (iDEF-PseRAAC (Zuo et al., 2019[60]) and DEFPRED (Kaur et al., 2021[30])) were functional during our manuscript preparation (accessed on 13 March 2022). 

iDEF-PseRAAC is a multi-class SVM predictor coupled with RAAA descriptor. iDEF-PseRAAC is freely available at http://bioinfor.imu.edu.cn/idpf/public/. In the case of the iDEF-PseRAAC web server, the query sequence pertains to five defensin families, including insect defensins, invertebrate defensins, plant defensins, vertebrate defensins and unclassified defensins. 

DEFPRED, on the other hand, is an *in silico* method developed for the prediction and design of defensins. The web server provides users with two options for the prediction of the query sequence: (i) discriminating defensins from AMPs (obtained from Model 1) and (ii) discriminating defensins from any random protein sequences (obtained from Model 2). In particular, Model 1 and Model 2 were trained using SVM-based models coupled with the 50 and 60 selected important features, respectively (Kaur et al., 2021[30]). Moreover, DEFPRED is able to predict potential defensins from primary protein sequences, but the length of the query sequence should be in the range of 10 to 60 residues. Furthermore, the web server provides users with a protein scan module that plays an important role in identifying specific regions in a protein. These regions can contribute in the designing of defensins. The DEFPRED web server is freely available at https://webs.iiitd.edu.in/raghava/defpred/.

## Shortcomings of Existing Predictors and Future Perspectives for Improving the Prediction Performance

To date, there are six ML-based predictors in existence that have been developed to predict defensins and their family/subfamily as summarized in Table 1(Tab. 1). These ML-based predictors could effectively facilitate the prediction of the defensin family/subfamily and exhibit promising predictive performance. However, several shortcomings remain in these approaches that need to be addressed to develop more accurate and useful models as summarized hereafter.

First, the existing training datasets were relatively limited, especially for the defensin family and subfamily. Thus, the prediction performance of the defensin family and subfamily was not satisfying for real-life applications. In the future, when more defensin family and subfamily become available, more samples should be gathered and then employed to train a more comprehensive model (Charoenkwan et al., 2021[6], 2022[7]; Kabir et al., 2022[27]).

Second, all the existing ML-based predictors were trained on the training datasets with high homologous sequences based on the CD-HIT threshold of 0.8-1.0 (Table 2(Tab. 2)). It could be stated that iDPF-PseRAAAC (Zuo et al., 2015[61]) and iDEF-PseRAAC (Zuo et al., 2019[60]) could achieve promising prediction performances when these two models were evaluated based on the dataset having high sequence identity. On the other hand, the prediction performance for those models was unsatisfactory based on the dataset having low sequence identity. Therefore, in order to construct a high-quality dataset, the CD-HIT threshold should be set as 0.3-0.4 to avoid over-estimation of the model's performance (Dao et al., 2019[18]; Feng et al., 2019[22]; Lai et al., 2019[32]; Lv et al., 2019[40]; Su et al., 2018[49]; Xu et al., 2019[57]).

Third, almost all of the existing ML-based predictors, including PseRAAAC (Zuo et al., 2015[61]), iDEF-PseRAAC (Zuo et al., 2019[60]) and DEFPRED (Kaur et al., 2021[30]), were developed based on a black-box computational method (SVM) (Table 1(Tab. 1)). Motivated by this limitation, our group has proposed the scoring card method (SCM) which is a simple and interpretable model (Charoenkwan et al., 2013[15]; Huang et al., 2012[25]). The SCM method has been effectively applied to characterize and predict a variety of biological activities of proteins and peptides (Charoenkwan et al., 2021[8][9], 2020[10][11][13][16][17], 2013[15]; Huang et al., 2012[25]). The main contribution of the SCM method can be described into two major aspects: (i) the SCM method outperforms the well-known SVM and RF methods in terms of simplicity and interpretability. Specifically, this method identifies desired proteins using only the weighted sum between the composition and propensity scores and (ii) the SCM method provides propensity scores for 20 amino acids and 400 dipeptides that could help to provide insight into the characteristics of the proteins and peptides. Another solution for overcoming the limitations of the black-box method is to make use of the Shapley Additive explanation (SHAP) algorithm (Lundberg and Lee, 2017[39]). The SHAP approach can provide both, the feature importance scores and the directionality of features.

Fourth, there is a lack of comprehensive assessment of the state-of-the-art feature encoding methods and ML algorithms in the prediction of defensins and their family/subfamily. Nevertheless, this comprehensive assessment provided could serve and facilitate users to select appropriate feature encodings/ML algorithms and provide useful guidelines for the development of more accurate and robust models in the future (Li et al., 2021[34]; Liang et al., 2021[36]).

Finally, as described in many articles (Basith et al., 2020[3]; Charoenkwan et al., 2021[6], 2022[7][14]; Kabir et al., 2022[27]), user-friendly web servers are considered as useful tools that are able to identify defensins and their family/subfamily without the use of experimental evidence. Although there are six computational approaches in existence, only two of them (i.e., iDEF-PseRAAC (Zuo et al., 2019[60]) and DEFPRED (Kaur et al., 2021[30])) were deployed as freely available web servers for the prediction of defensins and their family/subfamily.

## Conclusions

In this study, we have conducted a comprehensive review and assessment of six current state-of-the-art computational approaches for predicting defensins and their family/subfamily in terms of different important aspects, covering the dataset quality, feature encoding methods, feature selection schemes, ML algorithms, cross-validation methods and web server availability/usability. In addition, we performed a comparative analysis of the existing computational approaches for predicting defensins and their family/subfamily. For the prediction of defensins, DEFPRED outperforms Karnik's method in terms of generalization ability, robustness and utility. In case of the defensin family and subfamily prediction, iDEF-PseRAAC outperforms ID_RAAA and iDPF-PseRAAAC in terms of their predictive performance and robustness. These computational approaches are able to facilitate the identification of defensins and their family/subfamily. However, several shortcomings remain in these approaches that need to be addressed. Herein, five crucial aspects to develop more accurate and useful models have been listed as follows: (i) compiling an up-to-date dataset, (ii) excluding highly homologous sequences, (iii) using an interpretable method, (iv) performing a comprehensive assessment of the state-of-the-art feature encoding methods and (vi) constructing a web server. We anticipate that this comprehensive review will provide useful guidance to researchers interested in developing cutting-edge computational approaches for the prediction of defensins and their family/subfamily.

## Declaration

### Ethical statement

This review paper does not include animal or human experiments.

### Conflicts of interest

The authors declare no conflict of interest.

### Author contributions statement

WS: Conceptualization, project administration, supervision, investigation, manuscript preparation and revision. PC: Data analysis; data interpretation, investigation and manuscript preparation. NS: Manuscript revision. SMHM, OT and NS: Manuscript preparation. All authors reviewed and approved the manuscript.

### Acknowledgments

This work was fully supported by the College of Arts, Media and Technology, Chiang Mai University, and partially supported by the Chiang Mai University and Mahidol University. In addition, computational resources were supported by the Information Technology Service Center (ITSC) of Chiang Mai University.

## Figures and Tables

**Table 1:**
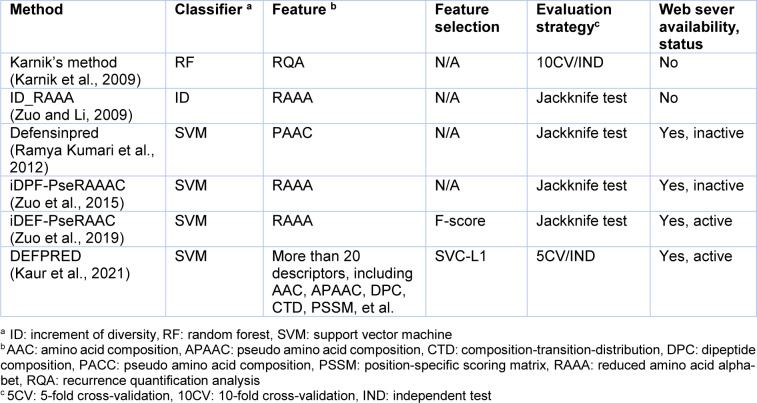
A list of currently available machine learning-based methods for the predictions of defensins and their family/subfamily

**Table 2:**
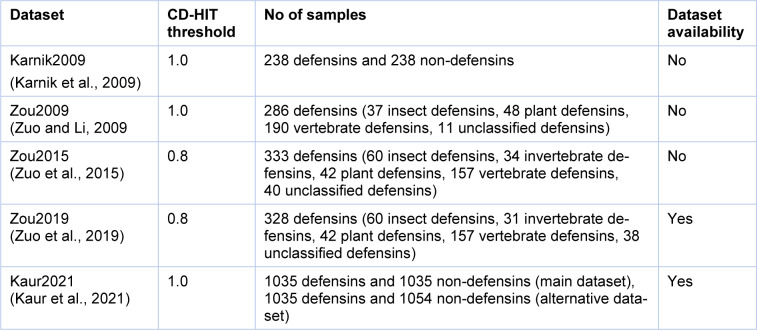
The detailed information of the existing datasets used for analyzing in this review

**Table 3:**
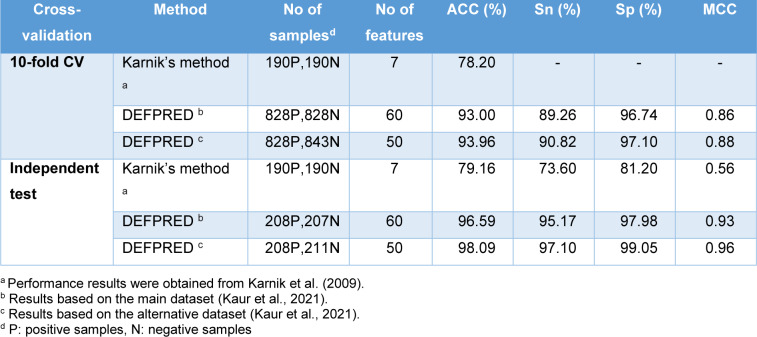
Performance comparison of Karnik's method and DEFPRED for the prediction of defensins

**Table 4:**
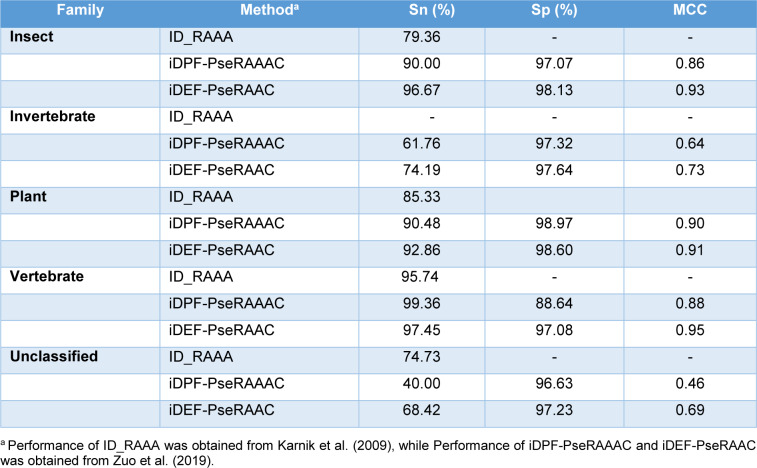
Performance comparison of ID_RAAA, iDPF-PseRAAAC and iDEF-PseRAAC for the prediction of defensins family

**Table 5:**
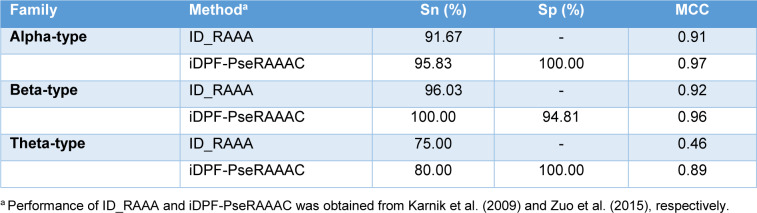
Performance comparison of ID_RAAA and iDPF-PseRAAAC for the prediction of vertebrate defensins subfamily.
